# Gelatin methacrylate hydrogel scaffold carrying resveratrol-loaded solid lipid nanoparticles for enhancement of osteogenic differentiation of BMSCs and effective bone regeneration

**DOI:** 10.1093/rb/rbab044

**Published:** 2021-07-30

**Authors:** Bangguo Wei, Wenrui Wang, Xiangyu Liu, Chenxi Xu, Yanan Wang, Ziqi Wang, Jinnuo Xu, Jianzhong Guan, Pinghui Zhou, Yingji Mao

**Affiliations:** Department of Orthopedics, First Affiliated Hospital, Bengbu Medical College, Bengbu 233004, China; Anhui Province Key Laboratory of Tissue Transplantation, Bengbu Medical College, Bengbu 233030, China; School of Life Sciences, Bengbu Medical College, Bengbu 233030, China; Anhui Province Key Laboratory of Translational Cancer Research, Bengbu Medical College, Bengbu 233030, China; Department of Orthopedics, First Affiliated Hospital, Bengbu Medical College, Bengbu 233004, China; School of Life Sciences, Bengbu Medical College, Bengbu 233030, China; Department of Orthopedics, First Affiliated Hospital, Bengbu Medical College, Bengbu 233004, China; Anhui Province Key Laboratory of Tissue Transplantation, Bengbu Medical College, Bengbu 233030, China; School of Life Sciences, Bengbu Medical College, Bengbu 233030, China; Department of Orthopedics, First Affiliated Hospital, Bengbu Medical College, Bengbu 233004, China; Anhui Province Key Laboratory of Tissue Transplantation, Bengbu Medical College, Bengbu 233030, China; Department of Orthopedics, First Affiliated Hospital, Bengbu Medical College, Bengbu 233004, China; Department of Orthopedics, Fuyang People‘s Hospital, Fuyang 236000, China; Department of Orthopedics, First Affiliated Hospital, Bengbu Medical College, Bengbu 233004, China; Anhui Province Key Laboratory of Tissue Transplantation, Bengbu Medical College, Bengbu 233030, China; School of Life Sciences, Bengbu Medical College, Bengbu 233030, China

**Keywords:** gelatin methacrylate, bone marrow mesenchymal stem cells, solid lipid nanoparticles, resveratrol, bone regeneration

## Abstract

Critical-sized bone defects caused by traumatic fractures, tumour resection and congenital malformation are unlikely to heal spontaneously. Bone tissue engineering is a promising strategy aimed at developing *in vitro* replacements for bone transplantation and overcoming the limitations of natural bone grafts. In this study, we developed an innovative bone engineering scaffold based on gelatin methacrylate (GelMA) hydrogel, obtained via a two-step procedure: first, solid lipid nanoparticles (SLNs) were loaded with resveratrol (Res), a drug that can promote osteogenic differentiation and bone formation; these particles were then encapsulated at different concentrations (0.01%, 0.02%, 0.04% and 0.08%) in GelMA to obtain the final Res-SLNs/GelMA scaffolds. The effects of these scaffolds on osteogenic differentiation of bone marrow mesenchymal stem cells (BMSCs) and bone regeneration in rat cranial defects were evaluated using various characterization assays. Our *in vitro* and *in vivo* investigations demonstrated that the different Res-SLNs/GelMA scaffolds improved the osteogenic differentiation of BMSCs, with the ideally slow and steady release of Res; the optimal scaffold was 0.02 Res-SLNs/GelMA. Therefore, the 0.02 Res-SLNs/GelMA hydrogel is an appropriate release system for Res with good biocompatibility, osteoconduction and osteoinduction, thereby showing potential for application in bone tissue engineering.

## Introduction

Bone is a fundamental motor organ of vertebrates that not only provides structural support for the body but also acts as a protective scaffold for internal organs [1, 2]. Approximately 3–5% of adult human bones are dissolved and renewed every day; this process plays an important role in regulating serum calcium and phosphorus levels [1, 3, 4]. Therefore, bone exhibits a strong regenerative ability; however, critical-sized bone defects caused by traumatic fractures, tumour resection and congenital malformation are unlikely to self-heal [5–7]. Common clinical treatments for large bone defects include various types of bone grafts including autografts, allografts and artificial bone grafts [8–10]. All types of bone grafts show serious disadvantages [4, 7]. For instance, artificial bone is costly to produce, whereas allogeneic bone carries a risk of pathogen transmission and immune rejection. Even autologous bone graft, the gold standard of bone transplantation, presents some limitations such as a risk of infection, donor site pain and long surgical procedures [11, 12]; in addition, because of the low tissue availability, it is difficult to meet clinical needs. Therefore, new substitutes for natural bone grafts must be identified for use in clinical practice.

Bone tissue engineering is a promising strategy aimed at developing *in vitro* bone replacements for transplantation and overcoming the issues of natural bone grafts [13–15]. These bone graft substitutes are grown from cellular seeds in controlled environments that include efficient scaffolds, abundant seed cells and growth factors [16]. Particularly, the scaffolds play a pivotal role in maintaining mechanical stability compared with the adjacent host bone, while also supporting cellular activity and guiding bone regeneration [17–19]. Gelatin methacrylate (GelMA) is a photo-crosslinked and modified hydrogel that has attracted considerable attention because of its cell adhesion properties, which are conferred by gelatin [20–26]. This material is widely used in bone tissue engineering because of its good biocompatibility and osteoconduction ability [27–29]. For instance, Lin *et al.* [30] cultured bone marrow mesenchymal stem cells (BMSCs) with different concentrations of GelMA hydrogel (7.5%, 10% and 15%) and found that the osteogenic differentiation of BMSCs in 10% GelMA was higher than that in the two other groups [30]. Shao *et al.* [27] encapsulated bone morphogenetic protein-2-coated nano-hydroxyapatite in GelMA hydrogel to fabricate bone tissue engineering scaffolds to repair bone defects. Although GelMA hydrogels are promising elements for bone tissue engineering, their deficiency in osteoinduction must be compensated by using osteogenic drugs.

Resveratrol (Res, 3,4’,5-trihydroxystilbene) is a natural phytoestrogen found in various plants [31]. Several studies have reported the beneficial biological effects of Res, stemming from its anti-aging, antioxidant, antitumour and anti-inflammatory actions [32–36]. Recent reports have highlighted the properties of Res in promoting osteogenic differentiation and bone formation [37–41]. For instance, Zhao *et al.* [42] found that Res improved osteoblast formation in a dose-dependent manner at concentrations up to 20 μM. Furthermore, Choi *et al.* [43] demonstrated that Res promoted bone regeneration in critical-sized calvarial defects. However, the poor solubility and significant anticancer action of Res at high concentrations may lead to serious effects such as uncontrolled drug release and high biological toxicity [36, 44]. Therefore, an appropriate drug release system must be established for Res.

Solid lipid nanoparticles (SLNs) are submicron colloidal carriers that present numerous advantages, such as ensuring good solubility and stability of the enclosed drugs, as well as high biocompatibility and bioavailability [45, 46]. Based on these properties, SLNs have received increased attention and are widely used to treat various diseases, such as cancer, Alzheimer’s and hypertension, among others [47–51]. Our previous study showed that Res-loaded SLNs (Res-SLNs) inhibited the proliferation, invasion and migration of human breast cancer cells [36]. In addition, SLNs have been reported for their ability to provide sustained release of drugs [52, 53]. For instance, Wang *et al.* [46] demonstrated that the sustained release of curcumin from SLNs was ∼50% after 4 days in a shaker. Moreover, Teskac and Kristl [54] observed slow and sustained release of ∼75% Res from the shell of SLNs over 7 days. However, the retention time of Res-SLNs was very short when the nanoparticles were injected directly into the bone defect site.

**Figure 1.**
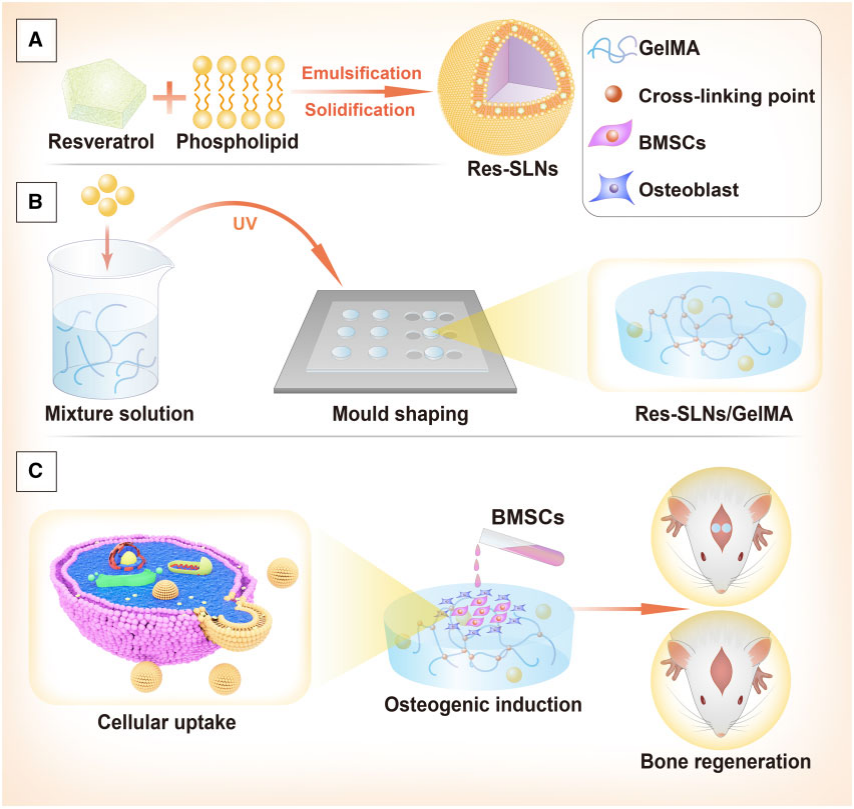
Schematic illustration of Res-SLNs/GelMA hydrogel scaffolds for bone regeneration. (**A**) Synthesis of Res-SLNs. (**B**) Preparation of Res-SLNs/GelMA. (**C**) Scaffolds promote osteogenesis differentiation of BMSCs cultured on the scaffolds and facilitate bone regeneration in rat cranial defects

To overcome these disadvantages, we used a GelMA hydrogel as a carrier to encapsulate Res-SLNs and evaluated the influence of different concentrations of Res-SLNs incorporated in GelMA (Res-SLNs/GelMA) hydrogel scaffolds on the osteogenic differentiation of BMSCs and bone regeneration *in vivo* (Fig. 1). Our results demonstrate the ability of an optimized Res-SLNs/GelMA scaffold to promote the osteogenic differentiation of BMSCs and bone regeneration, making this system a promising candidate for use in bone tissue engineering.

## Materials and methods

### Reagents

Res (99%) was obtained from Aladdin (Shanghai, China). Polyoxyethylene (40) stearate (Myrj 52) and dimethyl sulfoxide were purchased from Sigma-Aldrich (St Louis, MO, USA). Stearic acid and chloroform were obtained from Sinopharm Chemical Reagent Co., Ltd. (Shanghai, China). Lecithin, ethylene diamine tetraacetic acid (EDTA), bovine serum albumin (BSA), 4′,6-diamidino-2-phenylindole (DAPI) and 1% Alizarin Red S (ARS) solution at pH 4.1 were obtained from Solarbio Co. (Beijing, China). Cell counting kit-8 (CCK-8) and 4% paraformaldehyde were purchased from Biosharp (Shanghai, China). An osteogenic induction medium was obtained from Cyagen (Guangzhou, China). The alkaline phosphatase (ALP) staining kit was obtained from Yeasen (Shanghai, China). Osteocalcin (OCN) primary antibody, CD31 primary antibody and Cy3-labelled secondary antibody were obtained from Affinity Biosciences (Jiangsu, China). HiScript II QRT SuperMix for quantitative PCR (qPCR) and ChamQ SYBR Colour qPCR Master Mix were obtained from Vazyme (Nanjing, China). Dulbecco’s Modified Eagle’s Medium/Hams F12 (DMEM/F12, 1:1), foetal bovine serum and penicillin-streptomycin were obtained from Gibco (Grand Island, NY, USA). The GelMA hydrogel was obtained from EFL (Suzhou, China). All other chemicals were of analytical grade.

### Synthesis of Res-loaded SLNs

Res-SLNs were synthesized according to our previously reported methods [36]. Briefly, 200 mg of Myrj 52 was added to 30 ml of ultrapure water. Subsequently, the organic phase, consisting of 150 mg of Res, 200 mg of stearic acid and 100 mg of lecithin in 10 ml of chloroform, was injected into the aqueous phase at 75°C ± 2°C under agitation at 1000 rpm over 40 min to completely evaporate the chloroform. Next, 10 ml of cold water (4°C) was injected into the condensed aqueous/organic mixture, and the flask was transferred on ice under continuous stirring for 2 h. After centrifugation at 20 000 rpm (Avanti J25 centrifuge, JA 25.50 rotor; Beckman Coulter, Brea, CA, USA), the resulting suspension was lyophilized. In parallel, SLNs were fabricated using the same protocol without the addition of Res. The nanoparticles were sterilized by ultraviolet radiation for 5 min (Fig. 1).

### Characterization of Res-loaded SLNs

The surface morphology of SLNs and Res-SLNs was examined using a JEM-1230 transmission electron microscope (JEOL, Tokyo, Japan) at an accelerating voltage of 120 kV. The crystalline structures of Res, SLNs and Res-SLNs after freeze-drying were analysed using an X-ray diffractometer (D8 Advance; Bruker, Billerica, MA, USA). The patterns were scanned over a 2θ range of 10°–60° at 40 kV and 25 mA and analysed using Origin 8.0 software (OriginLab Corporation, USA). The particle size and zeta potential were determined via photon correlation spectroscopy (Zetasizer Nano ZS, Malvern Instruments, Malvern, UK) at 25°C ± 5°C. The percent entrapment rate of Res was determined using a UV-Vis spectrophotometer. Briefly, the weight of Res in the Res-SLNs was denoted as *W*_R_. The weight of total Res-SLNs was recorded as *W*_R__–__S_. The drug loading efficiency (*W*_E_) was denoted as *W*_E_ = *W*_R_/*W*_R__−__S_ × 100%.

### Preparation of Res-loaded SLNs/GelMA scaffolds

Based on previous studies, GelMA hydrogel was prepared at a concentration of 10% by adding 1 g GelMA and 0.05 g photo-initiator into 10 ml phosphate-buffered saline (PBS). Next, the GelMA prepolymer solution was filtered through a 0.22-μm filter. Next, Res-SLNs of different weights were added to the sterilized 10% GelMA hydrogel and ultrasonically mixed for 10 s to obtain Res-SLNs/GelMA solutions of different concentrations (0.01%, 0.02%, 0.04% and 0.08% w/v). The Res-SLNs/GelMA scaffolds were obtained after exposure to UV light (83 mW/cm^2^, 405 nm) for 30 s. The SLNs/GelMA scaffolds were prepared with 0.05% blank SLNs. All scaffolds were immersed in PBS for 12 h before further experiments.

### Characterization of Res-loaded SLNs/GelMA scaffolds

The section morphologies of the lyophilized scaffolds were observed by scanning electron microscopy (GeminiSEM 300, Carl Zeiss, Oberkochen, Germany). The Young’s moduli of scaffolds with a height of 5 mm and diameter of 3 mm were measured using a universal mechanical testing machine (E10000, Instron, Norwood, MA, USA) at a compression rate of 2 mm min^−1^ and distance of 1.2 mm. The water contact angle (WCA) of the lyophilized scaffolds was observed using a WCA analyser (DSA25S, Data Physics, Stuttgart, Germany). The different scaffolds were soaked in 2 U/ml of collagenase II at 37°C to investigate hydrogel degradation *in vitro* as described in our previous study [55].

### Determination of Res release from hydrogel scaffolds *in vitro*

To investigate the release rate of Res from SLNs/GelMA scaffolds, 3 ml of 0.08% Res-SLNs/GelMA hydrogel was added to a bottle containing 0.1 L PBS (0.01 M, pH 7.4) after photo-crosslinking. Subsequently, the bottle was sealed and shaken at 37°C and 100 rpm. At each time point (0.5, 1, 4, 7, 10, 13, 16, 19, 22, 25 and 28 days), 1 ml supernatant was sampled, and ethanol was added to bring the total sample volume to 2 ml for demulsification. This mixture was shaken, centrifuged at 15 000 rpm, and filtered through a 0.45-μm filter. The released Res was quantified using a UV-Vis spectrophotometer by measuring the absorption at 304 nm. The release rate of Res from GelMA scaffolds was measured by applying the same method to 0.08 wt% Res/GelMA scaffolds.

### Isolation and culture of BMSCs

BMSCs were obtained from a 4-week-old male Sprague–Dawley (SD) rat (Shushan Laboratory Animal Centre, Hefei, China) as previously described [56]. Briefly, bilateral femora and tibias were harvested, and epiphyses were cut-off. The bone marrow was extracted with 5 ml DMEM/F12 using a syringe. The isolated marrow cells were suspended and incubated in DMEM/F12 containing 10% foetal bovine serum and 1% penicillin/streptomycin at 37°C in a 5% CO_2_ atmosphere. The medium was replaced every 3 days. Cells from passages two to three were used in the experiments.

### Viability of BMSCs on different scaffolds

Live/dead staining was conducted to measure cell viability. Briefly, 1 × 10^5^ BMSCs/well in a 2-ml culture medium were cultured on 6-well culture plates coated with 1 ml hydrogel. After 3 days of culture, the samples were stained using a calcein-AM/propidium iodide double stain kit according to the manufacturer’s instructions. Finally, the cells were observed using a fluorescence inverted microscope.

In addition, the CCK-8 assay was used to assess cell viability after 1 and 3 days of culture. First, 100 μl of BMSC culture medium was seeded into 100-μl hydrogel-coated 96-well plates at a density of 5 × 10^3^ cells/well. At different time points, 100 μl of complete medium containing 10% CCK-8 solution was added to each well. After incubation at 37°C in a 5% CO_2_ atmosphere for 3 h, the supernatant was transferred into new plates. The optical density (OD) value was measured at 450 nm using a microplate reader. The cell viability was denoted as cell viability (%) = (OD_experiment—_OD_blank_)/(OD_control—_OD_blank_) × 100%.

### Osteogenesis measurement of BMSCs on Res-loaded SLNs/GelMA scaffolds *in vitro*

To evaluate osteogenic differentiation, 2 × 10^5^ BMSCs in 2 ml DMEM/F12 complete medium were seeded into 6-well plates that had been pre-treated with 1 ml of different hydrogel scaffolds. After 2 days of culture, the medium was changed to 2 ml of osteogenic differentiation medium and refreshed every 3 days.

#### ALP activity

ALP activity, an early marker of osteogenesis, was verified by ALP staining. After 7 days of osteogenic induction, we conducted ALP staining using a staining kit according to the manufacturer’s instructions. Briefly, the cells were fixed for 90 s, followed by staining with nitro-blue tetrazolium chloride/5-bromo-4-chloro-3-indolyl phosphate (BCIP/NBT) in the dark. After 15 min incubation, the superabundant BCIP/NBT reagent was removed. The blue ALP-positive cells were observed using an inverted microscope.

#### Determination of calcium content

ARS staining was performed to further analyse osteogenic differentiation. After 14 days of osteogenic induction, the cells were fixed with 4% paraformaldehyde for 20 min and then stained with 1% ARS solution at pH 4.1. After 5 min of incubation, the superabundant ARS solution was removed, and the cells were rinsed with deionized water.

#### OCN immunofluorescence staining

The expression of OCN, a late marker of osteogenic differentiation, was measured via immunofluorescence staining. After 14 days of osteogenic culture, the samples were fixed using 4% paraformaldehyde, permeabilized with 0.3% Triton X-100, and blocked using 1% BSA. The cells were incubated in 5 μg/ml OCN primary antibody at 4°C overnight and stained using a Cy3-labelled secondary antibody in the dark, after which DAPI was used to detect the appearance of nuclei.

#### RT-PCR analysis

After 14 days of osteogenic culture, total RNA was extracted from BMSCs using TRIzol. Subsequently, the HiScript II Q RT SuperMix kit was used to synthesize cDNA according to the manufacturer’s instructions. A Real-Time System and ChamQ SYBR qPCR Master Mix were used for real-time qPCR (RT-qPCR). Relative mRNA expression was normalized to that of the housekeeping gene β-actin and computed using the 2^−^^ΔΔ^^*C*^^t^ method. All gene-specific primers used in this experiment are shown in Table 1.

**Table 1.** Quantitative RT-PCR primer sequences

Gene	Forward primer (5′–3′)	Reverse primer (5′–3′)
β-actin	CCCATCTATGAGGGTTACGC	TTTAATGTCACGCACGATTTC
*Alp*	GGACCCTGCCTTACCAACTC	GTGGAGACGCCCATACCATC
*Ocn*	CTCAACAATGGACTTGGAGCC	GGCAACACATGCCCTAAACG
*Runx2*	CCGAGACCAACCGAGTCATTTA	AAGAGGCTGTTTGACGCCAT
*Opn*	CCAGCCAAGGACCAACTACA	AGTGTTTGCTGTAATGCGCC

### Implantation of hydrogel scaffolds in rat calvarial critical-size defect model

All animal experiments were approved by the ethics committee of the medical faculty of Bengbu Medical College (Approval Number No. 2019100). Six-week-old male SD rats were purchased from Shushan Laboratory Animal Centre, Hefei, China. All rats were housed in a 12-h light/dark cyclic room and acclimatized with free access to food and water for 7 days before the surgical procedures.

The scaffolds (GelMA, SLNs/GelMA and Res-SLNs/GelMA) with a diameter of 5 mm and height of 1 mm were prepared by UV light irradiation. Forty SD rats were randomly divided into four groups. The rats were anesthetized by injecting 10% chloral hydrate solution into the abdomen. The hair on the head was shaved off, and the skin was sterilized using iodophor. A 1.5-cm incision was made at the midline of the calvarium. The scaffolds were implanted into two bilateral full-thickness 5-mm diameter defects created using a dental trephine drill. Next, the skin incisions were sutured. The control group had no implanted scaffolds.

### Effects of different hydrogel scaffolds on bone regeneration *in vivo*

#### Micro-computed tomography measurement

At 4 and 8 weeks after surgery, the rats were euthanized using a lethal dose of 10% chloral hydrate solution. Calvaria was collected and fixed in 4% paraformaldehyde for 24 h. The calvaria was scanned by micro-computed tomography (μ-CT) (SkyScan1176, SkyScan, Aartselaar, Belgium) operating at 65 kV and 385 mA and equipped with a 1-mm Al filter. The 3D reconstruction and analysis of bone volume over tissue volume (BV/TV) ratio were conducted using the associated software.

#### Histological analysis

The calvaria was soaked for 30 days in an EDTA decalcifying solution (pH 7.2), which was replaced once per day. The samples were dehydrated, hyalinized and embedded in paraffin; 4-μm-thick sections were cut at the central area of implantation, and the deparaffinized sections were stained with haematoxylin and eosin (H&E) and Masson’s trichrome reagents. The bone formation area was evaluated under an optical microscope.

#### Immunohistochemical analysis

The sections were dewaxed and rehydrated, placed in citrate buffer for antigen retrieval, exposed to 3% methanol/H_2_O_2_ for blocking endogenous peroxidase, and blocked with 3% BSA. The sections were then incubated with the primary antibody against OCN (Affinity Biosciences, 1:200) and horseradish peroxidase-conjugated secondary antibody. Next, diaminobenzidine and haematoxylin were used for staining and counterstaining of the nucleus.

#### Immunofluorescence staining

The sections were deparaffinized and rehydrated, placed in EDTA antigen retrieval buffer and blocked with 3% BSA. Next, the sections were incubated with the primary antibody and secondary antibody. DAPI was used for counterstaining of the nucleus. Finally, the sections were incubated with a spontaneous fluorescence quenching reagent and covered with an anti-fade mounting medium.

### Statistical analysis

All data were analysed using SPSS 20.0 software (SPSS, Inc., Chicago, IL, USA). Comparisons between groups were analysed by one-way analysis of variance, followed by Tukey’s post-hoc tests. A *P* values <0.05 was considered to indicate statistically significant results. All experiments were performed in triplicate.

## Results

### Characterization of Res-loaded SLNs

**Figure 2.**
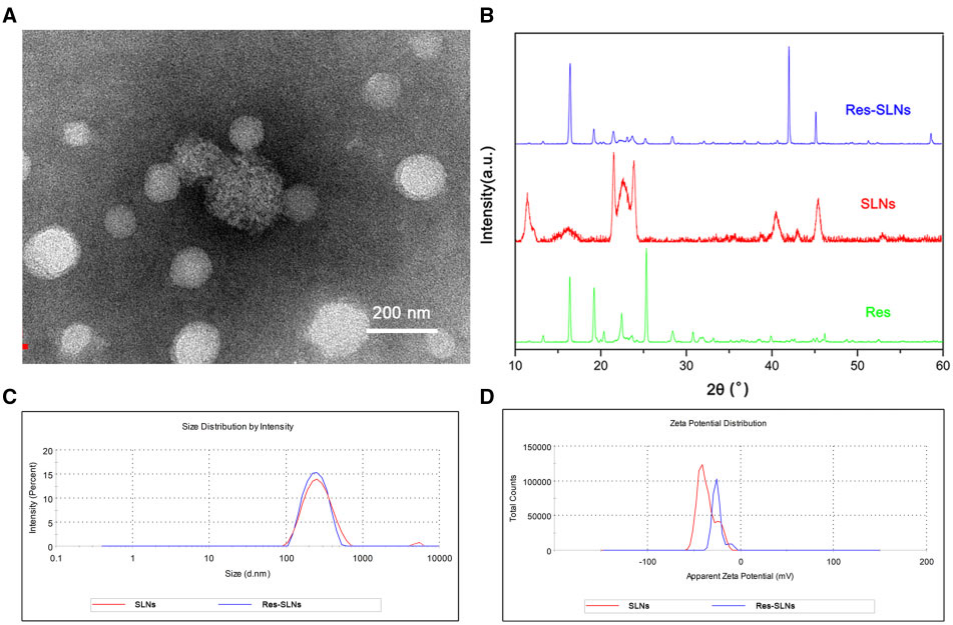
Characterization of Res-SLNs. (**A**) Transmission electron microscope photograph of Res-SLNs. (**B**) X-ray diffraction spectra for res, SLNs and Res-SLNs. (**C**) Particle size distribution of SLNs and Res-SLNs. (**D**) Zeta potential of SLNs and Res-SLNs

The morphology of Res-SLNs was investigated by transmission electron microscopy (Fig. 2A). The Res-SLNs were spherical, with a monomodal size distribution; no aggregation was observed. The X-ray powder diffraction patterns are presented in Fig. 2B. Diffraction peaks were observed at 16.39°, 19.22°, 22.44° and 25.34° in the Res pattern, indicating its highly crystalline structure. Similar peaks were detected in Res-SLNs, suggesting that Res was successfully incorporated into the SLNs. The average diameters of SLNs and Res-SLNs were ∼140 ± 39 nm (polydispersity index = 0.31 ± 0.01) and 150 ± 55 nm (polydispersity index = 0.23 ± 0.01), respectively (Fig. 2C). The zeta potentials of the SLNs and Res-SLNs were approximately −37.3 ± 0.9 and −26 ± 1 mV, respectively (Fig. 2D). The drug loading efficiency was calculated as 33.5% ± 1.2%.

### Characterization of hydrogel scaffolds

**Figure 3.**
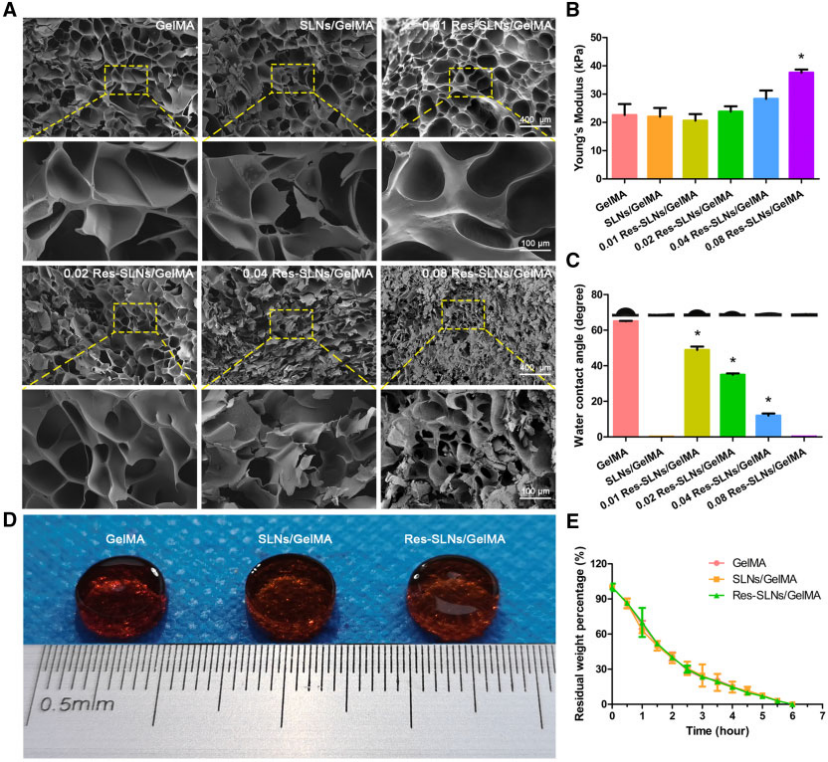
Characterization of Res-SLNs/GelMA hydrogel scaffolds. (**A**) Scanning electron microscope photograph, (**B**) Young’s modulus and (**C**) WCA of different hydrogel scaffolds. (**D**) The appearance of different hydrogel scaffolds (stained with eosin) after 2 h of degradation. (**E**) Degradation of different hydrogel scaffolds *in vitro*. (*n* = 3, *P <* *0.05 compared with control)

As shown in Fig. 3A, there was no significant difference in the pore size between the GelMA and SLNs/GelMA groups, indicating that the addition of SLNs did not affect the hydrogel pore size. However, compared with the GelMA group, the pore sizes of different Res-SLNs/GelMA groups gradually decreased with an increase in the Res content.

As shown in Fig. 3B, the Young‘s modulus of the GelMA scaffold was 22.6 ± 0.4 kPa. However, there was no significant difference in the Young’s moduli between the GelMA and SLNs/GelMA groups, indicating that the addition of SLNs did not affect the Young’s moduli of the hydrogels. The Young’s moduli of different Res-SLNs/GelMA scaffolds increased with an increase in the Res-SLN content. Particularly, the Young’s modulus of the 0.08 Res-SLNs/GelMA group was 37.6 ± 0.1 kPa, which was significantly higher than that of the GelMA group, suggesting that the addition of Res increased the Young’s modulus of the hydrogel scaffolds.

WCA is an important index used to measure the hydrophilicity of materials. As shown in Fig. 3C, the WCA of GelMA was 65.06° ± 0.25°. When compared with the GelMA group, the WCA of different Res-SLNs/GelMA samples decreased with increasing Res-SLN concentrations. Interestingly, the SLNs/GelMA group and sample 0.08 Res-SLNs/GelMA were extremely absorbent; water droplets were absorbed immediately after coming in contact with the scaffold surface, and the WCA was 0° when the quality of SLNs in the scaffolds reached 0.05%. This result indicates that the addition of SLNs improved the hydrophilicity of the scaffolds.

Scaffold biodegradability is a critical factor in bone tissue engineering. As shown in Fig. 3D and E, all hydrogel scaffolds were completely dissolved after 6 h of incubation. There was no significant difference in the degradation rate between the different hydrogel scaffolds, indicating that the addition of Res and Res-SLNs did not affect GelMA degradation. Moreover, the degradation rate of GelMA hydrogel decreased over time, suggesting that degradation begins at the hydrogel scaffold surface.

### *In vitro* release of Res from hydrogel scaffolds

**Figure 4.**
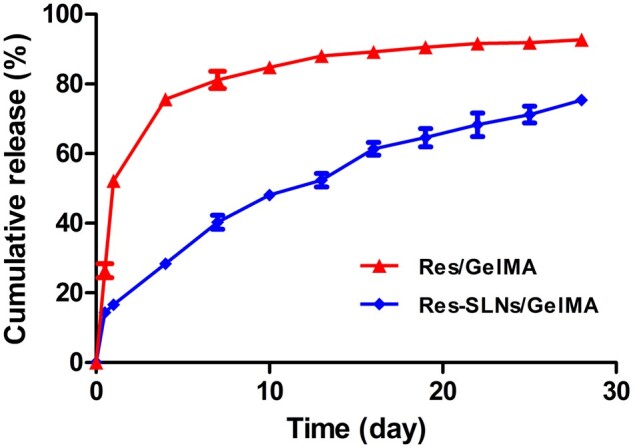
*In vitro* release profile of Res from Res/GelMA and Res-SLNs/GelMA scaffolds

As shown in Fig. 4, the cumulative release of Res in the Res/GelMA group reached 75% after 4 days. However, in the Res-SLNs/GelMA group, the rate of drug release gradually slowed after the 14% burst release at 0.5 days, and the cumulative release amount was ∼75% at 28 days, indicating that SLNs/GelMA have an excellent sustained-release effect. To avoid the toxic effect of burst release on BMSCs, the hydrogel scaffolds used in the subsequent experiments were first soaked in PBS for 12 h at 37°C.

### Effect of hydrogel scaffolds on the viability of BMSCs

**Figure 5.**
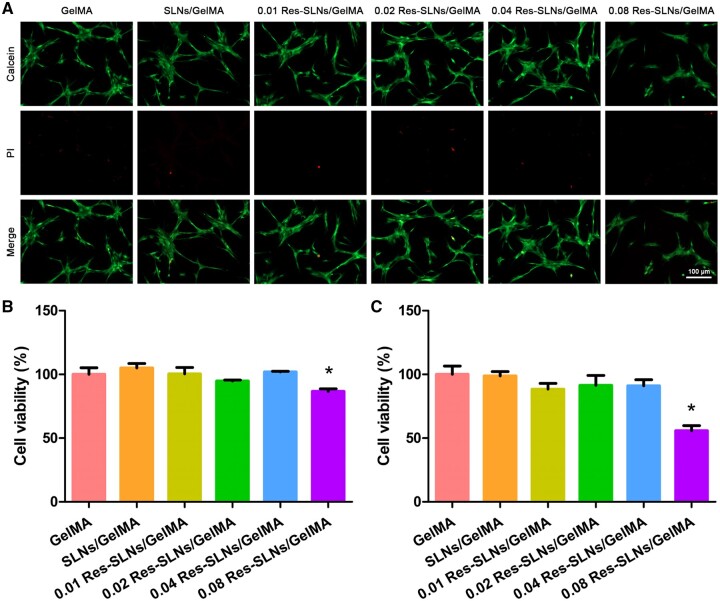
Cell viability assay. (**A**) Live/dead staining. CCK-8 assay after (**B**) 1 day and (**C**) 3 days of BMSCs cultured on different hydrogel scaffolds. (*n* = 3, *P <* *0.05 compared with control)

As shown in Fig. 5A, there was no significant difference in the number of red-stained dead cells and green-stained live cells between the SLNs/GelMA group and GelMA group, indicating that the addition of SLNs did not affect BMSC activity. However, the number of living cells in the 0.08 Res-SLNs/GelMA hydrogel scaffold group was significantly reduced. The results of the CCK-8 assay also showed that the addition of low-concentration Res-SLNs to the hydrogel scaffold had no specific effect on BMSCs after 1 and 3 days of culture, whereas the viability of BMSCs decreased significantly when the concentration reached 0.08% (Fig. 5B and C).

### Effect of hydrogel scaffolds on the osteogenic differentiation of BMSCs *in vitro*

**Figure 6.**
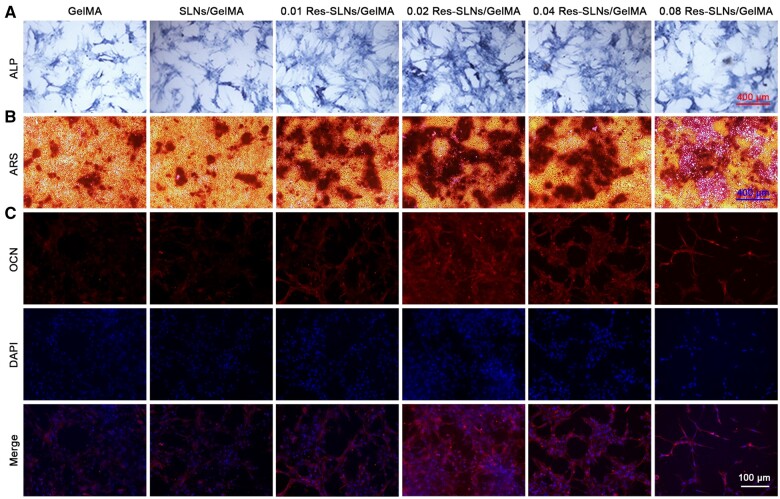
Osteogenesis differentiation. (**A**) ALP staining for 7 days. (**B**) ARS staining for 14 days. (**C**) OCN immunofluorescence staining for 14 days after incubation in osteogenic medium

The possible promoting effect of Res-SLNs/GelMA scaffolds on osteogenic differentiation was investigated by evaluating ALP activity, calcium content and OCN levels in BMSCs. As shown in Fig. 6A, no significant difference in the ALP-positive cell count was observed between the GelMA and SLNs/GelMA groups, indicating that the addition of SLNs to the hydrogel scaffolds did not affect early osteogenic differentiation. However, ALP activity in all Res-SLNs/GelMA scaffolds was higher than that in the GelMA group; particularly, ALP activity was strongest in the 0.02 Res-SLNs/GelMA group. These results indicate that Res-SLNs/GelMA scaffolds promoted early-stage osteogenic differentiation, specifically in the case of 0.02 Res-SLNs/GelMA scaffolds. Similar results were obtained via ARS staining and OCN immunofluorescence staining. These suggest that the addition of Res-SLNs into GelMA scaffolds enhanced late-stage osteogenic differentiation, with the best-promoting effect observed in the 0.02 Res-SLNs/GelMA scaffolds (Fig. 6B and C).

**Figure 7.**
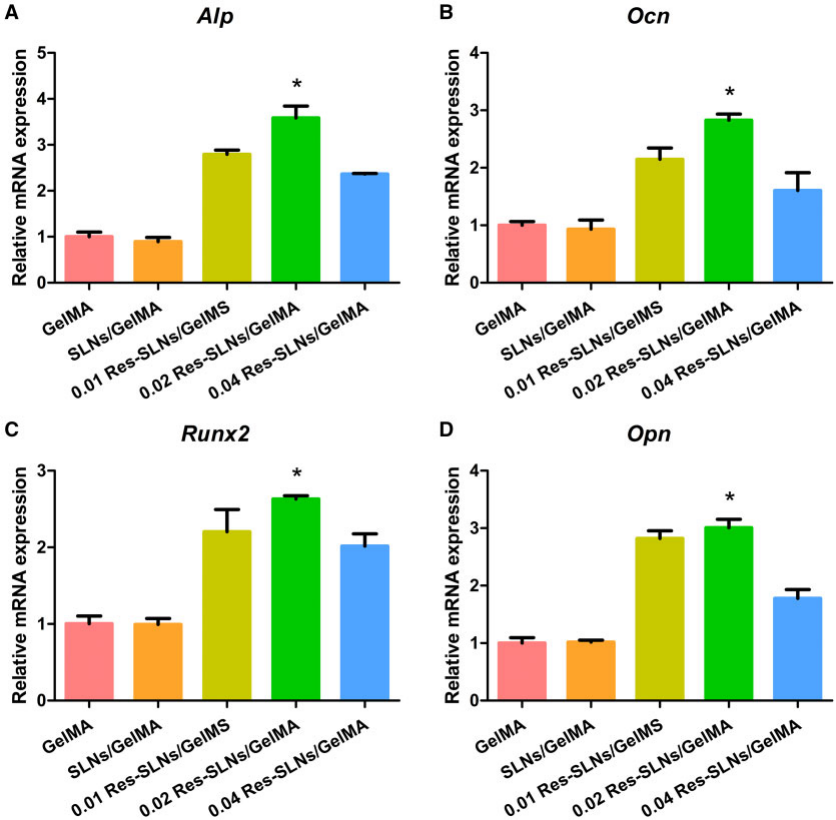
mRNA expression of osteogenic genes (**A**) *Alp*, (**B**) *Ocn*, (**C**) *Runx2* and (**D**) *Opn* quantified using RT-qPCR after 14 days of osteogenic induction. (*n* = 3, *P <* *0.05 compared with other groups)

We also performed RT-qPCR to quantify the expression of osteogenic genes, including *Alp*, *Ocn*, *Runx2* and *Opn*, and investigate the influence of Res-SLNs/GelMA on osteogenic differentiation. The expression of the *Alp* in the GelMA group was similar to that in the SLNs/GelMA group, indicating that the addition of SLNs did not affect the expression of this gene (Fig. 7). In contrast, *Alp* expression in the Res-SLNs/GelMA groups increased significantly compared with that in the GelMA group, with 0.02 Res-SLNs/GelMA presenting the highest expression. Similar patterns were observed for the expression of *Ocn*, *Runx2* and *Opn*, suggesting that Res-SLNs/GelMA scaffolds promoted the expression of osteogenic genes, with the optimal concentration of Res-SLNs being 0.02%. Therefore, 0.02 Res-SLNs/GelMA scaffolds were used in subsequent animal studies *in vivo*.

### Effect of hydrogel scaffolds on bone regeneration *in vivo*

**Figure 8.**
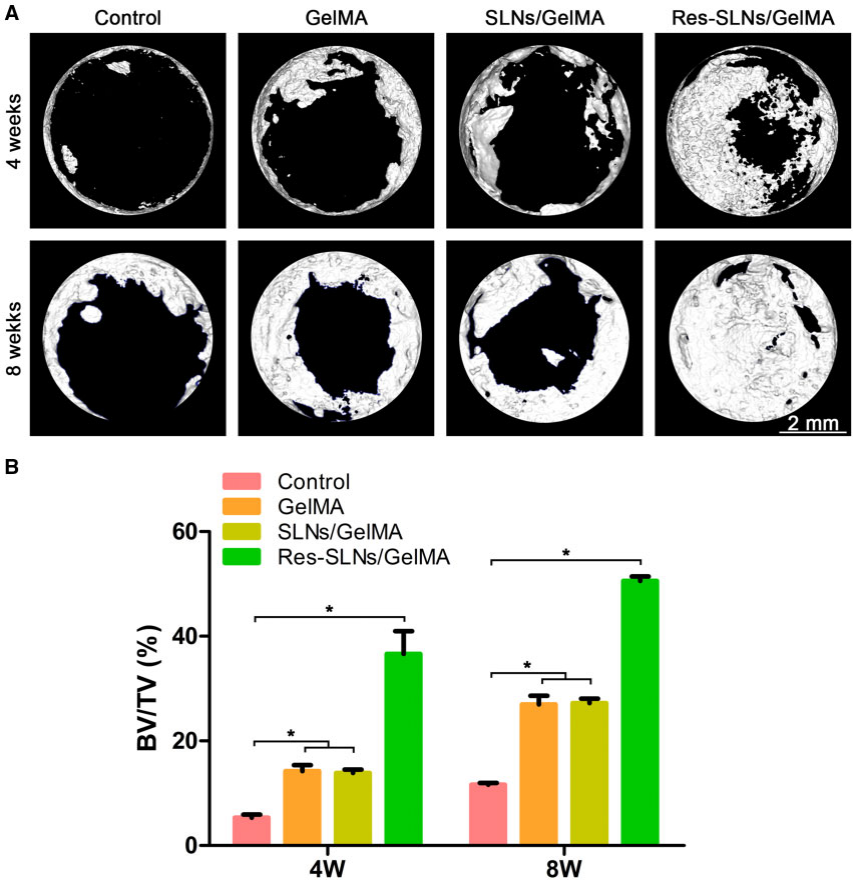
Imaging analysis. (**A**) μ-CT scan of the samples of calvarial defects in the rat. (**B**) Quantification of BV/TV (*n* = 3, *P <* *0.05 compared with other groups)

We performed μ-CT to evaluate whether Res-SLNs/GelMA scaffolds facilitated bone regeneration (Fig. 8A). After a 4-week post-operation period, only a small amount of new bone tissue was observed in the control group. However, a more substantial formation of new bone tissue was observed in the other groups compared with the control group, with the Res-SLNs/GelMA group exhibiting the highest bone regeneration rate. Quantitative analysis showed that the BV/TV ratio in Res-SLNs/GelMA was significantly higher than that in other groups (Fig. 8B). At 8 weeks after surgery, this trend became more evident; the defect site was completely covered by new bone tissue in the Res-SLNs/GelMA group, and the BV/TV ratio reached 50%.

**Figure 9.**
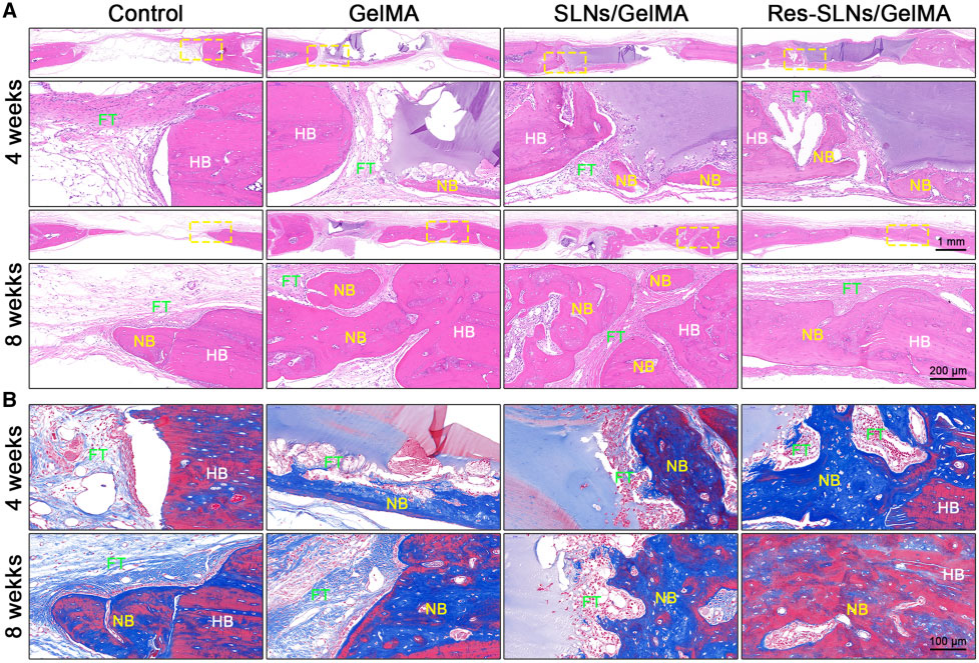
Histological staining. (**A**) H&E staining and (**B**) Masson’s trichrome staining of de-paraffin sections. NB, newly formed bone tissue; HB, host bone; FT, fibrous tissue

The skull samples were then decalcified and prepared into paraffin sections. The newly formed bone tissue was evaluated by H&E staining and Masson’s trichrome staining, and the results were consistent with those of μ-CT (Fig. 9A and B). At 4 weeks after skull defect modelling, the GelMA hydrogel was still observed in each scaffold group; new bone tissue had grown at the site of the degraded hydrogel, indicating that the hydrogel scaffolds had suitable osteoconduction ability and a degradation rate that matched osteogenesis. At 8 weeks post-operation, the GelMA hydrogel in the Res-SLNs/GelMA group was completely degraded, newly formed bone tissue had entirely covered the defect site, the thickness had reached ∼80% of normal bone tissue, and the degree of bone tissue maturity was significantly higher than that in the other groups, where large numbers of bone lacunae were observed.

**Figure 10.**
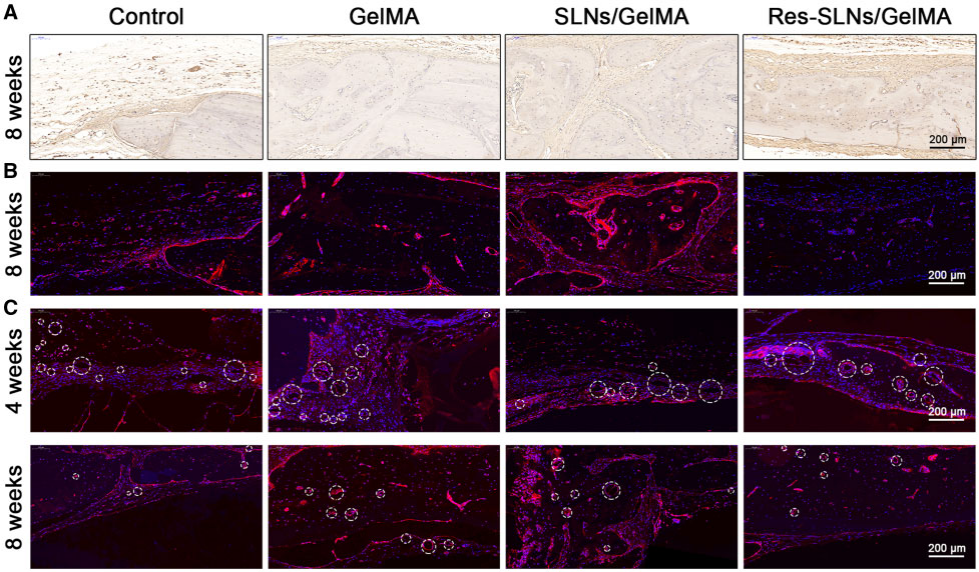
Immunological staining. (**A**) Immunohistochemical staining of OCN. Immunofluorescence staining of (**B**) RUNX2 and (**C**) CD31. Circles indicate new blood vessels

The results of OCN immunohistochemical staining and RUNX2 immunofluorescence staining, shown in Fig. 10A and B, were consistent with those of H&E staining. Positive expression (stained as brown) in other groups was significantly higher than that in the control group, with the Res-SLNs/GelMA group showing the highest expression. Consistently, our results showed that Res-SLNs/GelMA hydrogel scaffolds very effectively promoted bone regeneration. CD31 is a marker of neonatal endothelial cells; hence, we performed CD31 immunofluorescence staining to measure neovascularization in each group (Fig. 10C). At 4 weeks after the operation, only a few CD31-positive vascular lumens with small diameters were observed in the bone defect of the control group. In contrast, in the other scaffold groups, a larger number of CD31-positive endothelial cells was observed; the cells were surrounded by round lumens, and a large number of vascular lumens with larger diameters were visible. However, the number of new vessels significantly decreased in the scaffold groups after 8 weeks post-surgery.

## Discussion

Bone has a strong ability to regenerate, and small bone defects can typically heal spontaneously [5, 6]. However, large bone defects caused by traumatic fractures, congenital malformations and resection of bone tumours may not heal normally and often require bone transplantation for repair [7–10]. Bone tissue engineering is a promising strategy aimed at developing bone graft substitutes *in vitro* and overcoming the defects of natural bone grafts [13, 14]. The key benefit of tissue engineering scaffolds is that it allows coordinating a drug release system with natural bone formation, to control the release of drugs or factors related to osteogenesis following the bone regeneration process [15–17]. It has been reported that Res, a natural phytoestrogen, can promote osteogenic differentiation and bone formation [37–40]. In this study, we designed and prepared Res-SLNs/GelMA scaffolds based on the sustained-release effect of SLNs and GelMA hydrogel. Through long-term slow release from the scaffolds, the concentration of Res at the defect area was maintained at the necessary rate to promote osteogenesis and repair large bone defects.

Characterization analysis verified that Res-SLNs were successfully synthesized using emulsification and low-temperature solidification methods and that the Res-SLNs/GelMA scaffolds exhibited good porosity and mechanical properties. Release tests *in vitro* showed excellent sustained release of Res from Res-SLNs/GelMA scaffolds, which was mainly related to the specific drug loading method [52]. The drug loading capacity of SLNs can be described by considering three different locations: surface adsorption, insertion in the shell, and core enrichment [54, 57]. Drug molecules located on the SLN surface were the main origin of the observed burst release. The smooth and continuous release following the initial burst originated from diffusion through the lipid matrix of the SLNs [46]. In addition, encapsulating the drug in the GelMA hydrogel contributed to the slow release [7].

An ideal bone graft scaffold should exhibit good biocompatibility [17]. The biocompatibility of new bone tissue scaffolds is typically tested by analysing their cytotoxicity, proliferation and differentiation [18, 19]. We evaluated the cytotoxicity of different hydrogel scaffolds. No evident effect of the addition of SLNs was observed on the toxicity of GelMA hydrogels, possibly because of the good physiological properties and biocompatibility of the SLN components [36]. In addition, different Res-SLNs/GelMA scaffolds affected the apoptosis of BMSCs. Res has been described as an anti-aging nutraceutical, that decreases apoptosis through the FAS/FAS-ligand pathway and other related biochemical activity [32]. However, the 0.08 Res-SLNs/GelMA scaffolds significantly reduced the viability of BMSCs; this agrees with our previous study, in which a high concentration of Res (IC_50_ = 72.06 ± 7.85 μg/ml) significantly inhibited the proliferation of breast cancer cells [36].

To evaluate the effect of Res-SLNs/GelMA scaffolds on the osteogenesis differentiation of BMSCs *in vitro*, we investigated the expression of ALP and calcium deposition, respectively. The results indicated that adding SLNs into the scaffolds affected early osteogenic differentiation, whereas Res-SLNs increased ALP expression after 7 days of osteogenic induction culture. Moreover, the 0.02 Res-SLNs/GelMA scaffolds promoted the production of calcium nodules at the late stage. Dai *et al.* [41] cultured BMSCs with different Res concentrations and found that Res promoted the differentiation of BMSCs into osteoblasts in a dose-dependent manner at concentrations up to 10 μM. The drug release test showed that the daily release of Res from SLNs/GelMA scaffolds during the first 16 days was ∼3% after the initial burst release. Hence, the concentration of Res in the culture media was ∼ 9 μM, which is consistent with previous studies.

To determine the influence of Res-SLNs/GelMA scaffolds on osteogenic differentiation, we measured the expression of genes *Alp* and *Runx2*, which are early indices of osteoblastic differentiation, as well as *Ocn* and *Opn*, which are late markers of bone maturation. After 14 days of osteo-induced differentiation, the fluorescence intensity of OCN in the 0.02 Res-SLNs/GelMA scaffolds was higher than that in the others. Because of the cytotoxicity of 0.08 Res-SLNs/GelMA, the number of BMSCs cultured on the scaffold was insufficient to satisfy RT-qPCR requirements. Therefore, RT-qPCR was performed only on the 0.01, 0.02 and 0.04 Res-SLNs/GelMA scaffolds. The mRNA expression of *Alp* and *Runx2* in BMSCs was significantly enhanced by the addition of Res-SLNs compared with the GelMA and SLNs/GelMA groups; the highest expression was recorded for the 0.02 Res-SLNs/GelMA sample, consistent with the previous ALP activity assay results. In addition, similar mRNA expression results were observed for *Ocn* and *Opn*, supporting the results obtained for OCN.

Subsequently, we implanted different scaffolds in cranial defect sites of SD rats to examine their effects on bone regeneration *in vivo*. At 4 and 8 weeks post-operation, the skulls were harvested and scanned by μ-CT. When compared with the control group, the amount of newly formed bone tissue and BV/TV in the scaffolds had both significantly increased, which may be related to the osteoconductivity of the GelMA hydrogel reported in previous studies [27, 28]. For instance, Xin *et al.* [29] prepared regenerated periosteum with 20% GelMA and found that the amount of new bone tissue in all hydrogel groups was significantly higher than that in the blank group. The mechanical properties of GelMA hydrogels can be affected by many factors, such as amino substitution rate, concentration and crosslinking time [20–22]. For instance, the biocompatibility of GelMA decreased with an increase in the degree of substitution, whereas the mechanical properties improved. Lin *et al.* [30] cultured BMSCs in different GelMA hydrogels at various concentrations (7.5%, 10% and 15%) and found that the osteogenic differentiation in 10% GelMA was significantly higher than that in the other two groups. Therefore, GelMA hydrogel was used at a concentration of 10% in this study.

The skull samples were decalcified and made into paraffin sections. The H&E and Masson’s staining assays showed similar results as μ-CT. In addition, the site containing degraded hydrogel was filled with newly formed bone tissue, indicating a suitable biodegradation rate of the hydrogel scaffolds matching the osteogenesis rate. GelMA hydrogel was prepared from methacrylic anhydride and gelatin [22]. The latter contains the target motifs of matrix metalloproteinase and arginine-glycine-aspartic acid, which may have played a role in improving cell adhesion and biodegradability [23–25]. Because <5% MA was used to synthesize GelMA, the bioactivity of matrix metalloproteinase and arginine-glycine-aspartic acid was not significantly affected [26].

Furthermore, OCN immunohistochemical staining and RUNX2 and CD31 immunofluorescence staining of sections were performed to evaluate bone regeneration. Similar trends were observed in OCN and RUNX2 staining assays, supporting the above results. Analysis of CD31 indicated that the GelMA scaffolds enhanced angiopoiesis at 4 weeks post-surgery, possibly because of the increased porosity of the hydrogel with degradation. Druecke *et al.* [58] investigated the influence of scaffolds with different pore sizes on neovascularization and found that scaffolds with large pores were the most suitable for the growth of new blood vessels. Angiogenesis was reported to peak at 2 weeks after the operation [29]. Subsequently, neovascularization began to shrink with the maturation of newly regenerated bone tissue [59]. This may explain why the expression of CD31 in all scaffolds decreased at 8 weeks post-surgery.

Collectively, our results indicate that Res-SLNs/GelMA hydrogel scaffolds can improve osteogenic differentiation *in vitro* and bone regeneration *in vivo*. Despite not performing ideally in the promotion of angiogenesis, our Res-SLNs/GelMA scaffolds show significant potential for application in bone tissue engineering. Further studies should focus on optimizing angiogenic properties.

## Conclusion

We successfully synthesized Res-SLNs using emulsification and low-temperature solidification methods. The Res-SLNs were then used to prepare Res-SLNs/GelMA hydrogels as bone tissue engineering scaffolds. Because of their architectures, the scaffolds achieved slow and sustained Res release and thereby enhanced osteogenic differentiation *in vitro* and bone regeneration *in vivo*. In addition, rat cranial defects sites implanted with Res-SLNs/GelMA hydrogel scaffolds were healed completely at 8 weeks post-operation. This study provides a potential therapeutic strategy for critical-sized bone defects.

## Funding

This work was supported by the Natural Science Foundation of Anhui Province (Grant No. 2008085QH362), Key Program of Anhui Educational Committee (Grant No. KJ2020ZD51), Translational Medicine Key Projects of Bengbu Medical College (Grant Nos. BYTM2019006 and BYTM2019012), Scientific Research Innovation Team of Bengbu Medical College (Grant No. BYKC201910) and 512 Talents Development Project of Bengbu Medical College (Grant Nos. by51202302 and by51202309).

*Conflict of interest statement*. None declared.
